# The causal relationship between gut microbiota and alopecia areata: a Mendelian randomization analysis

**DOI:** 10.3389/fmicb.2024.1431646

**Published:** 2024-07-12

**Authors:** Dezhao Bi, Jin Tong Tey, Dan Yao, Yutian Cao, Minyu Qian, Jianxin Shi, Shun Guo

**Affiliations:** ^1^Affiliated Hospital of Nanjing University of Chinese Medicine, Jiangsu Province Hospital of Chinese Medicine, Nanjing, China; ^2^The First Clinical Medical College of Nanjing University of Chinese Medicine, Nanjing, China; ^3^Nanjing University of Chinese Medicine, Nanjing, China

**Keywords:** alopecia areata, causal association, FinnGen, gut microbiota, Mendelian randomization

## Abstract

**Background:**

Increasing evidence suggests a robust correlation between the gut microbiome and alopecia areata. In light of the extensive diversity of gut microbiota, this study aims to utilize state-of-the-art and comprehensive data to explore the causative association between gut microbiota and alopecia areata.

**Objective:**

We conducted a Mendelian randomization (MR)-based two-sample study to elucidate the causal relationship between gut microbiota and alopecia areata.

**Method:**

Summary information on Ncase = 767 and Ncontrol = 394,105 cases of alopecia areata was obtained from the FinnGen study. A total of 473 gut microbial taxa were summarized from the genome-wide association study (GWAS) catalog. The study comprised a forward Mendelian randomization (MR) analysis with the gut microbiome as the exposure factor and alopecia areata as the outcome, as well as a reverse MR analysis with alopecia areata as the exposure factor and the gut microbiome as the outcome. Various analytical methods including inverse variance weighting (IVW), Weighted Median, MR-Egger, Weighted Mode, and Simple Mode were employed. Subsequently, sensitivity analysis was conducted to ensure the robustness of our research findings.

**Result:**

This study has established a causal relationship between gut microbiota and alopecia areata. Forward causal analysis revealed causality relationships between 16 gut microbial taxa and alopecia areata, while reverse causal analysis found that there may be a causal relationship between alopecia areata and 16 gut microbial taxa (not statistically significant).

**Conclusion:**

Our study findings suggest a causal relationship between gut microbiota and alopecia areata, providing potential guidance for future clinical trials.

## Introduction

Alopecia areata (AA) is a type of sudden hair loss that linked to autoimmune disorders and affects around 2% of the global population (Lee et al., 2020). It can manifest in different ways, from localized patches hair loss to total hair loss on the scalp and body (Pratt et al., 2017). The most common form is scalp patchy alopecia. Due to changes in appearance, it may have a detrimental impact on patients’ mental health and may potentially to trigger depression, anxiety, and other psychosocial issues (Muntyanu et al., 2023). At present, the exact cause of AA is not fully understood, but it is believed to involve the immune system attacking hair follicles and subsequent hair loss (Petukhova et al., 2010). Genetic and environmental factors are key influences on the development and progression of AA (Juárez-Rendón et al., 2017; Minokawa et al., 2022). In recent years, there has been growing acknowledge of the profound influence gut microbiota have on development and progression of diseases due to advancing medical knowledge and technology (Mahmud et al., 2022; Sánchez-Pellicer et al., 2022). With ongoing research, the study of gut microbiota has become a major field of study (Martin-Gallausiaux et al., 2021; Armet et al., 2022). Numerous diseases have been closely associated to gut microbiota, particularly in autoimmune diseases where their impact is increasingly recognized as prevalence rises. Recent studies have highlighted a direct connection between gut microbiota and certain autoimmune disorders, such as systemic lupus erythematosus and inflammatory bowel disease (Xu et al., 2022). Further investigation into the relationship between alopecia areata and gut microbiota is warranted to deepen our understanding in this area.

The gut microbiota is a diverse community of microorganisms in the human gastrointestinal tract, forming a symbiotic relationship with the body. These microorganisms engage in intricate and multifaceted interactions among themselves and with the host, aiding in the breakdown of dietary fiber, complex carbohydrates and other indigestible compounds for nutrient absorption (Zhao et al., 2019). They also play an important role in regulating intestinal barrier integrity to maintain homeostasis, supporting immune system development to prevent the occurrence of allergies and autoimmune diseases (Sittipo et al., 2018). They also producing beneficial metabolic products like vitamins, amino acids and fatty acids (Rowland et al., 2018). Overall, gut microbiota is crucial for various physiological processes including digestion, immunity regulation, pathogen protection as well as metabolism; thus serving as a key determinant for maintaining human health. Therefore understanding the link between alopecia areata and gut microbiota is essential for identifying potential treatment options. Given the unclear understanding of the precise pathological mechanisms behind AA, existing treatments like corticosteroids, other immunomodulators, and minoxidil offer limited efficacy while carrying notable side effects and high relapse rates. Therefore, investigating the correlation between AA and gut microbiota may lead to new developments in treatment and prevention strategies, enhancing our ability to prevent and treat AA. However, there has been relatively limited research on specific roles of gut microbiota in AA-related studies. Traditional observational studies are susceptible to biases due to numerous confounding factors during implementation. Hence we conducted an examination using existing summary data from genome-wide association studies (GWAS) results employing Mendelian randomization methods to investigate associations between gut microbiota composition and AA.

Mendelian randomization (MR) is a method that utilizes genetic variation [single nucleotide polymorphisms (SNPs)] as instrumental variables (IVs) to act as proxies for the exposure of interest This enables the investigation of causal relationships between exposure and specific outcomes (Larsson et al., 2023). It can effectively reduce the impact of confounding factors and reverse causality. Published genome-wide association studies have integrated associations between gut microbiota and SNPs, providing a convenient approach to assess the causal relationship between gut microbiota and the risk of alopecia areata. Therefore, this study employs the Mendelian randomization method to investigate the causal relationship between gut microbiota and alopecia areata from the perspective of genetic variation, aiming to provide new insights for the treatment and prevention of AA.

## Materials and methods

### Study design

**FIGURE 1:**
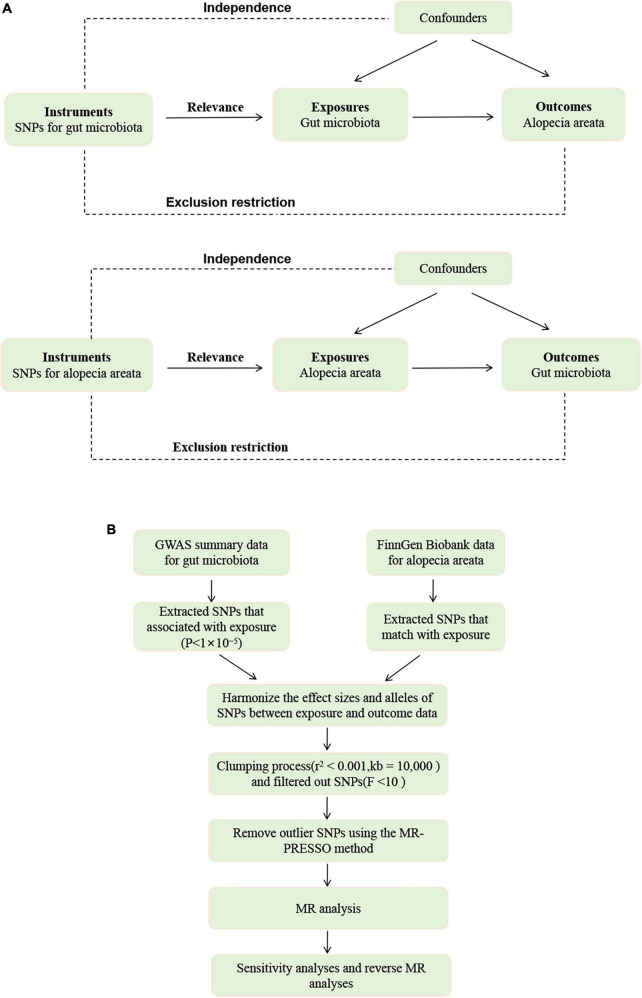
**(A)** The diagram of MR assumption. **(B)** The workflow of Mendelian randomization (MR) analysis.

To investigate the relationship between the 473 gut microbial taxa and alopecia areata, we employed a two-sample Mendelian Randomization study. We use genetic variations as instrumental variables (IVs) to obtain causal relationships. Therefore, all instrumental variables (IVs) must satisfy three assumptions: (1) IVs are significantly associated with exposure; (2) IVs affects the outcome solely through exposure; (3) There is no direct association between the IVS and the outcome. Gao et al. (2023) have provided the specifics of each in detail. Figure 1 and Supplementary Data Sheet depicts the MR design for this study.

### Date sources of exposure and outcome

#### GWAS data for gut microbiota

The comprehensive summary statistics for gut microbiota with genome-wide significant findings are openly accessible in the NHGRI-EBI GWAS Catalog, spanning from accession GCST90032172 to GCST90032644.^1^ Genetic variants associated with the gut microbiome were obtained from genome-wide association tests applied to 7,979,834 human genetic variants from the 5,959 individuals enrolled in the FINRISK 2002 cohort. This cohort is based on the Finnish population. Genome-wide association analysis of gut microbial taxa identifies 567 independent SNP-taxon associations (Qin et al., 2022).

#### GWAS data for alopecia areata

We also employed data summaries from the FinnGen Consortium^2^ in our analysis. In the FinnGen database (release R10, 2023), we selected alopecia areata (Ncase = 767, Ncontrol = 394,105). There are a total of 412,181 (230,310 females and 181,871 males) participants and 2,408 disease endpoints in the database for the European population. The FinnGen project provides a wealth of information about the participants, which can be accessed on the website.

### Genetic instrument selection

We applied a series of criteria to meticulously choose eligible genetic IVs: (1) Considering that the number of available IVs at *P* < 5 × 10^–8^ was quite limited, a loose cutoff of *P* < 1 × 10^–5^ was set to obtain a relatively large number of IVs (Fu et al., 2024); (2) To ensure the independence of instrumental variables, a clumping process was implemented with a condition of r^2^ < 0.001 and an LD window of 10 000kb. During this process, only the SNP with the lowest *P*-value was retained; (3) We assessed the robustness of the IVs for the selected SNPs by analyzing F-statistics and variance (R^2^) to mitigate the risk of instrumental variable bias [*F* > 10; F = R^2^ × (N-K-1)/K × (1-R^2^)] (Hu et al., 2022); and (4) when palindromic SNPs existed, the forward strand alleles were inferred using allele frequency information.

### Statistical analyses

In our MR study, we used five methods to robustly evaluate the causal relationship between gut microbiota and alopecia areata. The primary approach was utilized the inverse variance weighted (IVW) method, with MR Egger, Weighted Median, Weighted Mode, and Simple Mode serving as auxiliary methods (Hartwig et al., 2017; Xiang et al., 2023). We selected the IVW method as our primary approach due to of its ability to generate consistent causal effects in scenarios without heterogeneity (Mounier and Kutalik, 2023). If the IVW results are significant (*P* < 0.05), this confirms the presence of a causal relationship. The direction of the causal relationship can be determined by the estimated effect direction. The MR-Egger intercept test was specifically used to evaluate the presence of horizontal pleiotropy, with a significance level of *P* < 0.05 indicating its existence. Weighted Median allows for reliable causal inference even when up to 50% of IVs might be invalid, adding another layer of robustness to our analysis. The Simple Mode method determines the most commonly occurring causal estimate among the chosen SNPs, whereas the Weighted Mode calculates a weighted average that gives precedence to more robust IVs. When statistically significant results (*P* < 0.05) are obtained from the study analysis, it suggests a potential causal relationship between gut microbiota and alopecia areata.

### Sensitivity analysis

The variability among IVs was assessed through the application of Cochran’s Q statistic. A *P*-value of less than 0.05 signifies the presence of statistically significant heterogeneity among the IVs (Cohen et al., 2015). When the intercept term in the MR-Egger regression is close to zero, the magnitude of the horizontal multi-effect is smaller. If the result of the horizontal pleiotropic test is *P* > 0.05, it is considered that the horizontal pleiotropic does not exist (Burgess and Thompson, 2017). The MR-PRESSO method is used to identify and evaluate the impact of outliers. After detecting outliers, we will re-evaluate the causal effect after removing them (Verbanck et al., 2018). Additionally, we performed leave-one-out analysis, systematically excluding each SNP to assess the influence of individual SNPs (Zeng et al., 2023).

### Reverse Mendelian randomization analysis

To further assess the causal relationship between gut microbiota and alopecia areata, we conducted reverse MR analysis considering alopecia areata as the exposure and gut microbiota as the outcome, using the same methods and settings as the forward analysis. All statistical analyses were performed using R (version 4.3.3).

## Results

### Investigating the gut microbiota on alopecia areata

**FIGURE 2:**
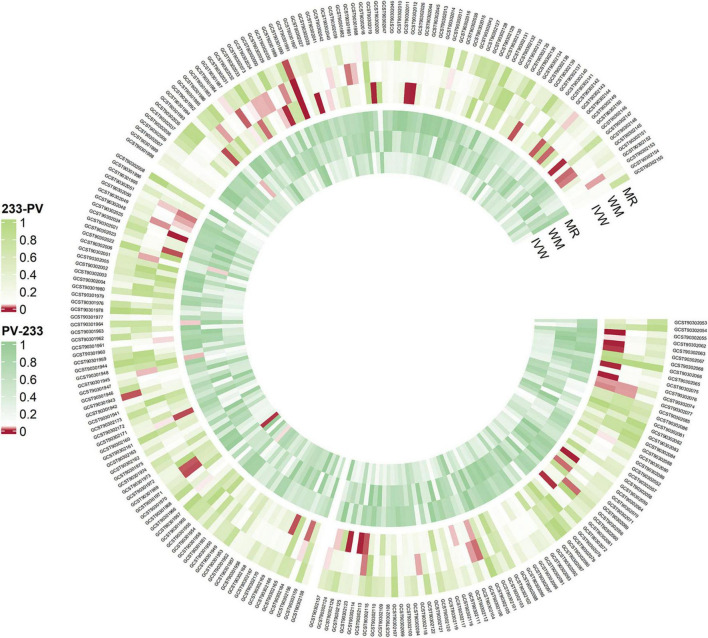
Overview of the causal role of gut microbiota and alopecia areata in MR analysis (outer section) and reverse MR analysis (inner section). The red color indicates statistical significance (*P* < 1 × 10^–5^). MR, MR-Egger method, WM, Weighted Median method, IVW, inverse variance weighted method.

**FIGURE 3:**
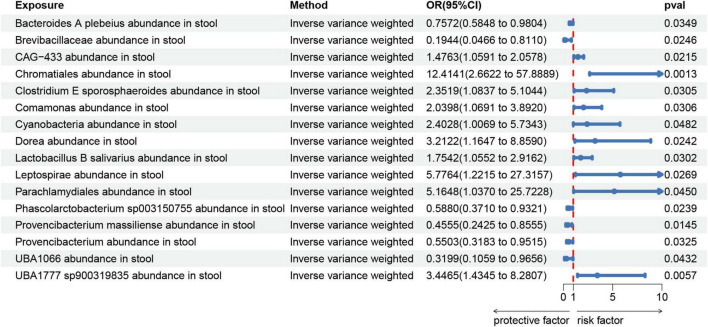
Forest plot shows the expression causality of gut microbiota for alopecia areata.

We created a circus plot to visually represent all of the data (Figure 2). We utilized IVW as the primary method (Supplementary Tables 1–6). Associated bacterial taxon (GTDB release 89 nomenclature). Our forward MR analysis, identified significant associations between 16 gut microbial taxa and AA (Figure 3). Specifically, there are 10 gut microbial taxa that may increase the risk of AA. *CAG-433* abundance in stool [OR = 1.4763, 95% CI: 1.0591–2.0578; *p* = 0.0215]; *Chromatiales* abundance in stool [OR = 12.4141, 95% CI: 2.6622–57.8889; *p* = 0.0013]; *Clostridium E sporosphaeroides* abundance in stool [OR = 2.3519, 95% CI: 1.0837–5.1044; *p* = 0.0305]; *Comamonas* abundance in stool [OR = 2.0398, 95% CI: 1.0691–3.892; *p* = 0.0306]; *Cyanobacteria* abundance in stool [OR = 2.4028, 95% CI: 1.0069–5.7343; *p* = 0.0482]; *Dorea* abundance in stool [OR = 3.2122, 95% CI: 1.1647–8.859; *p* = 0.0242]; *Lactobacillus B salivarius* abundance in stool [OR = 1.7542, 95% CI: 1.0552–2.9162; *p* = 0.0302]; *Leptospira* abundance in stool [OR = 5.7764, 95% CI: 1.2215–27.3157; *p* = 0.0269]; *Parachlamydiales* abundance in stool [OR = 5.1648, 95% CI: 1.037–25.7228; *p* = 0.045]; *UBA1777 sp900319835* abundance in stool [OR = 3.4465, 95% CI: 1.4345–8.2807; *p* = 0.0057].

**FIGURE 4:**
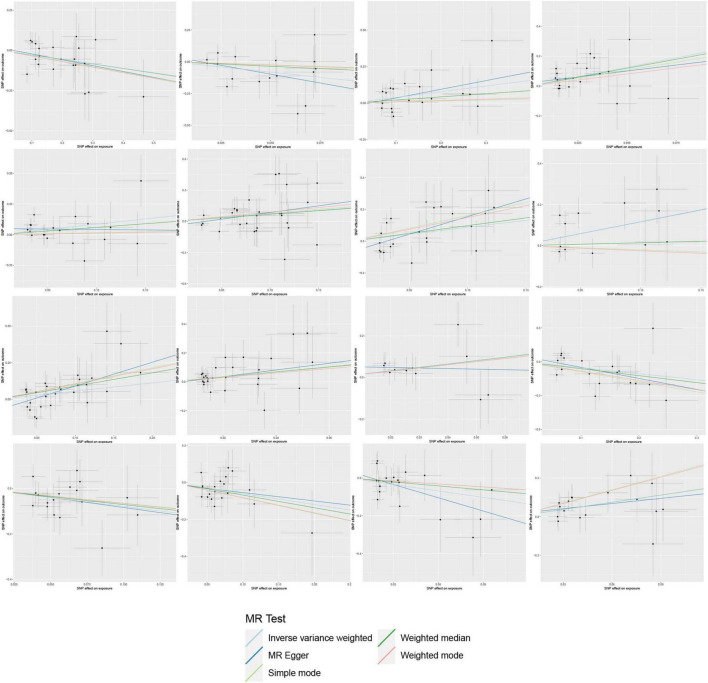
Scatter plots of the Mendelian randomization (MR) analysis.

**FIGURE 5:**
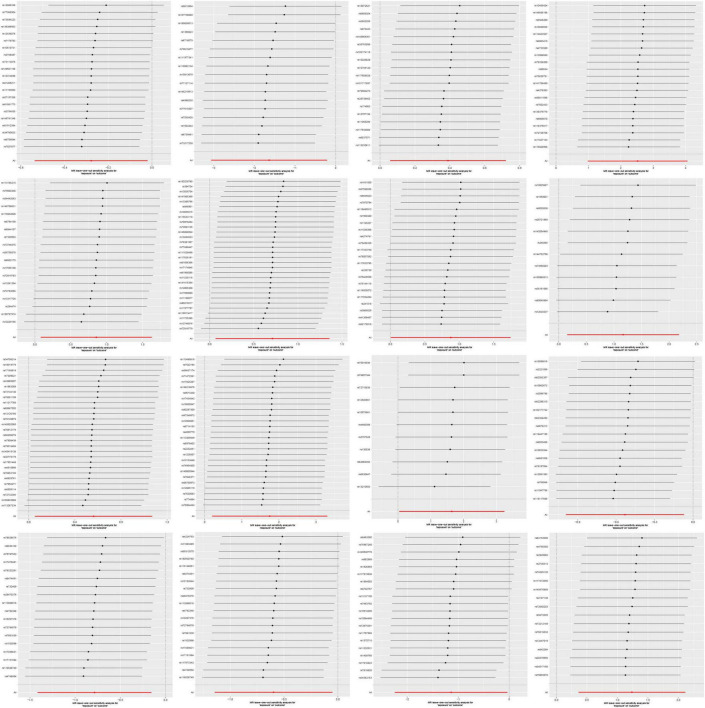
Leave-one-out diagrams for causal effects of gut microbiota on alopecia areata.

On the contrary, 6 gut microbial taxa exhibit a protective effect against AA. *Bacteroides A plebeius* abundance in stool [OR = 0.7572, 95% CI: 0.5848–0.9804; *p* = 0.0349]; *Brevibacillaceae* abundance in stool [OR = 0.1944, 95% CI: 0.0466–0.811; *p* = 0.0246]; *Phascolarctobacterium sp003150755* abundance in stool [OR = 0.5880, 95% CI: 0.371–0.9321; *p* = 0.0239]; *Provencibacterium massiliense* abundance in stool [OR = 0.4555, 95% CI: 0.2425–0.8555; *p* = 0.0145; *Provencibacterium* abundance in stool [OR = 0.5503, 95% CI: 0.3183–0.9515; *p* = 0.0325]; *UBA1066* abundance in stool [OR = 0.3199, 95% CI: 0.1059–0.9656; *p* = 0.0432]; Cochran’s Q test and MR-Egger regression intercept analysis revealed no significant heterogeneity or horizontal pleiotropy among the IVs. Scatter plots and leave-one-out analyses further corroborated our research findings (Figures 4, 5). Additionally, MR-PRESSO analysis indicated no detected outliers.

### Investigating the alopecia areata on gut microbiota

**FIGURE 6:**
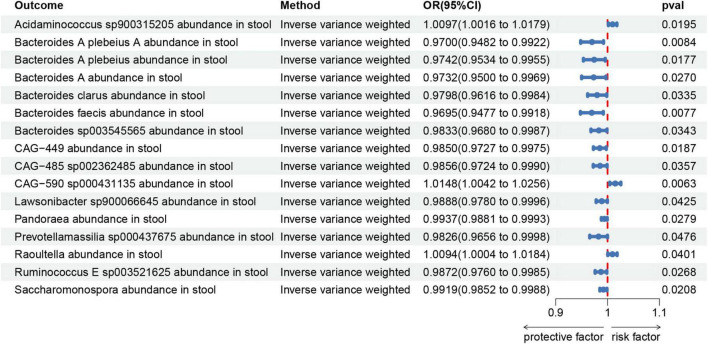
Forest plot shows the expression causality of alopecia areata for gut microbiota.

To assess the reverse causal impact of AA on 473 gut microbiota, two-sample reverse MR analyses were primarily conducted using the IVW method. As depicted in Figure 6.

**FIGURE 7:**
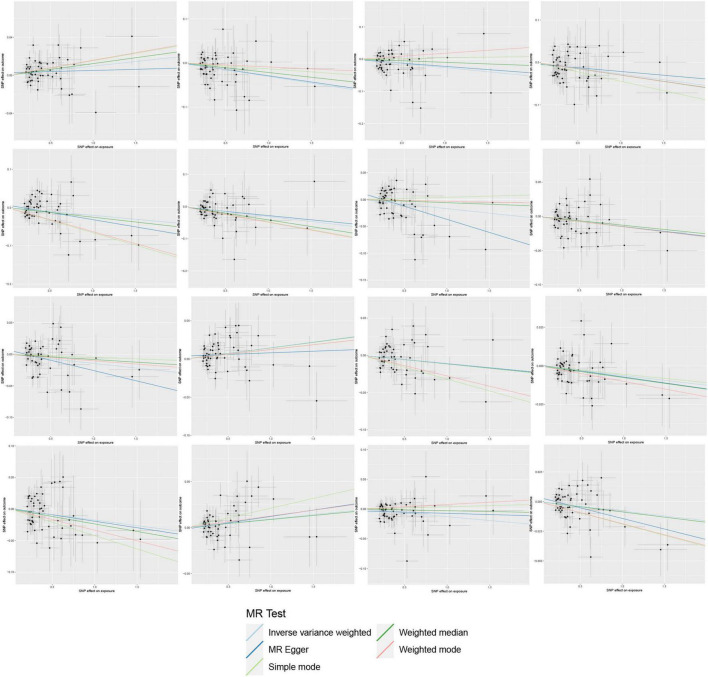
The reverse scatter plot results.

**FIGURE 8:**
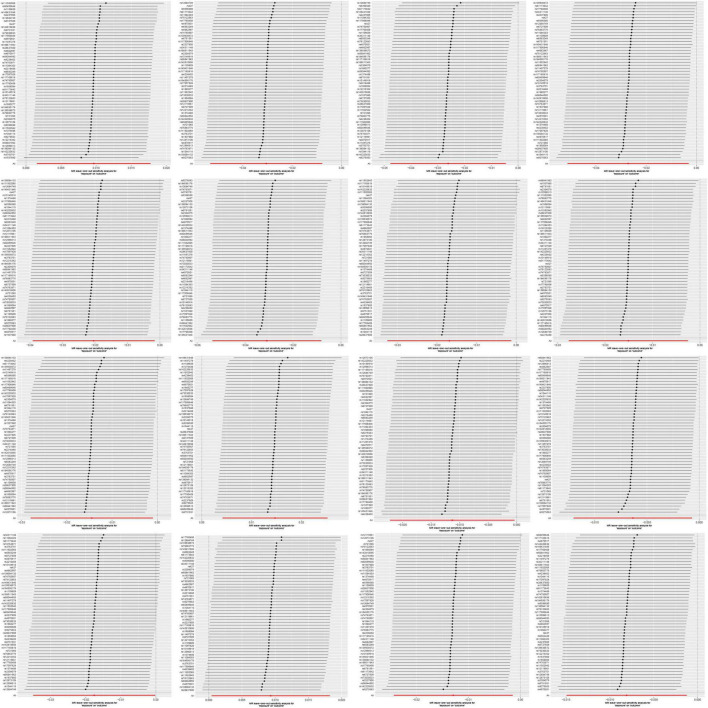
The reverse leave-one-out results.

In the reverse MR study, we also found potential causal relationships between AA and 16 gut microbial taxa. However, these causal relationships were not significant. Specifically: *Acidaminococcus sp900315205* abundance in stool [OR = 1.0097, 95% CI: 1.0016–1.0179; *p* = 0.0195]; *Bacteroides A plebeius A* abundance in stool [OR = 0.9700, 95% CI: 0.9482–0.9922; *p* = 0.0084]; *Bacteroides A plebeius* abundance in stool [OR = 0.9742, 95% CI: 0.9534–0.9955; *p* = 0.0177]; *Bacteroides A* abundance in stool [OR = 0.9732, 95% CI: 0.9500–0.9969; *p* = 0.027]; *Bacteroides clarus* abundance in stool [OR = 0.9798, 95% CI: 0.9616–0.9984; *p* = 0.0335]; *Bacteroides faecis* abundance in stool [OR = 0.9695, 95% CI: 0.9477–0.9918; *p* = 0.0077]; *Bacteroides sp003545565* abundance in stool [OR = 0.9833, 95% CI: 0.9680–0.9987; *p* = 0.0343]; *CAG-449* abundance in stool [OR = 0.9850, 95% CI: 0.9727–0.9975; *p* = 0.0187]; *CAG-485 sp002362485* abundance in stool [OR = 0.9856, 95% CI: 0.9724–0.9990; *p* = 0.0357]; *CAG-590 sp000431135* abundance in stool [OR = 1.0148, 95% CI: 1.0042–1.0256; *p* = 0.0063]; *Lawsonibacter sp900066645* abundance in stool [OR = 0.9888, 95% CI: 0.9780–0.9996; *p* = 0.0425]; *Pandoraea* abundance in stool [OR = 0.9937, 95% CI: 0.9881–0.9993; *p* = 0.0279]; *Prevotellamassilia sp000437675* abundance in stool [OR = 0.9826, 95% CI: 0.9656–0.9998; *p* = 0.0476]; *Raoultella* abundance in stool [OR = 1.0094, 95% CI: 1.0004–1.0184; *p* = 0.0401]; *Ruminococcus E sp003521625* abundance in stool [OR = 0.9872, 95% CI: 0.9760–0.9985; *p* = 0.0268]; *Saccharomonospora* abundance in stool [OR = 0.9919, 95% CI: 0.9852–0.9988; *p* = 0.0208]; Figure 7 illustrates the scatter graph, and Figure 8 displays the leave-one-out analysis results.

## Discussion

Although some studies have indicated a relationship between gut microbiota and autoimmune diseases, research on the connection between gut microbiota and AA remains relatively limited. Therefore, we leveraged publicly available genetic data to evaluate the relationship between 473 gut microbial taxa and alopecia areata. Our forward study confirmed causal effects of 16 specific gut microbial taxa on alopecia areata. In our reverse MR analysis, although the associations were not highly significant, alopecia areata may also exhibit causal relationships with 16 gut microbial taxa. We anticipate these findings to offer new potential targets for alopecia areata treatment and provide a theoretical foundation for future therapies.

A vast number of microorganisms, including fungi, viruses, and bacteria, inhabit our bodies, with most of them colonizing the gastrointestinal tract. The gastrointestinal tract is considered the body’s largest immune organ and plays a central role in regulating immune homeostasis. The colonization and stability of microorganisms in the gastrointestinal tract influence the development of the immune system and regulate immune mediators, thereby affecting the function of the intestinal barrier (Takiishi et al., 2017). However, dysbiosis of the microbiota can compromise the intestinal defense barrier, allowing for the translocation of harmful substances into the bloodstream, thereby triggering an immune response and increasing susceptibility to autoimmune diseases (Tie et al., 2023). Jia et al. (2023) found that the relative abundance of 14 bacterial species, such as *Lactobacillus salivarius*, *Bacteroides fragilis*, and *Clostridium bartlettii*, was significantly increased in patients with primary Sjögren’s syndrome and systemic lupus erythematosus. A molecular subgroup of *Bacteroides fragilis*, *Enterotoxigenic Bacteroides fragilis (ETBF)*, can secrete a specific gut toxin. This gut toxin disrupts the tight junctions of intestinal epithelial cells, increasing intestinal permeability and allowing intestinal contents to enter the bloodstream. This process activates and expands Th17 cells, which then secrete cytokines like IL-17, triggering local and systemic inflammatory responses. This may partly explain the pathogenic role of these microorganisms in autoimmune diseases (Chung et al., 2018; Cao et al., 2021).

Furthermore, gut microbiota play a crucial role not only in maintaining the intestinal barrier but also in significantly impacting the health of the skin barrier. Metabolites produced by gut microbiota can travel through the bloodstream to the skin, influencing the metabolism and immune function of skin cells. For instance, short-chain fatty acids synthesized by gut microbiota can regulate the function of regulatory T cells (Treg cells). Treg cells maintain immune system balance and stability by modulating the strength and direction of immune responses, which is essential for maintaining immune tolerance, preventing autoimmune diseases, and suppressing excessive immune reactions. Under normal conditions, Treg cells regulate immune responses by secreting inhibitory cytokines such as IL-10 and TGF-β, preventing autoimmune attacks. However, in patients with alopecia areata, this regulatory mechanism may fail, leading to an uncontrolled immune attack on hair follicles (Smith et al., 2013; Bertolini et al., 2020). The state of gut microbiota can influence the skin’s immune system, and vice versa. This interaction forms the so-called gut-skin axis, which is vital for maintaining the balance of the body’s immune system and skin health (Sinha et al., 2021).

Evidently, there is a distinct difference in the gut microbiota between alopecia areata patients and individuals without the condition (Lee et al., 2024). Therefore, studying the relationship between gut microbiota and alopecia areata is of great significance. Among them, *Bacteroides* accounts for a major part of the gut bacterial community. These Gram-negative anaerobic bacteria play various roles in the human gut microbiota and are major participants in maintaining the gut microbiota food web (Wexler, 2007). Although the presence of *ETBF* may be associated with the occurrence and development of certain inflammation-related diseases, Zafar and Saier (2021) argue that in the appropriate environment, the *Bacteroides genus* is a friend to humans. They can protect the intestine from pathogen invasion and provide nutrition to other microbiota within the intestine, thereby influencing the host’s health status (Zafar and Saier, 2021). Our findings are consistent with this perspective. In our study, we found that higher abundance of certain *Bacteroides species*, such as *Bacteroides A plebeius*, *Bacteroides clarus*, and others, may be associated with a lower risk of alopecia areata. In addition to the *Bacteroides genus*, Moreno-Arrones et al. (2020) found that specific gut microbiota, such as *Lachnospiraceae*, *Parabacteroides johnsonii*, and *Clostridiales vadin BB60 group*, are highly abundant in alopecia areata patients, while *Phascolarctobacterium succinatutens* and *Dorea longicatena* are significantly abundant in normal individuals (Moreno-Arrones et al., 2020). Xu et al. (2024) conducted a Mendelian randomization study and identified a relationship between 5 bacterial genera (*Butyricimonas*, *Enterorhabdus*, *Eubacterium (xylanophilum group)*, *Phascolarctobacterium*, and *Ruminococcaceae UCG003*) and AA. Among them, *Ruminococcaceae UCG003* was identified as a risk factor (Xu et al., 2024). The research findings indicate a significant association between gut microbiota and alopecia areata, further corroborating our study results. Our research findings also supplement previous studies. However, gut microbiota are complex and diverse, and currently, only a small portion of gut microbiota research is comprehensive. Furthermore, the relationship between gut microbiota and alopecia areata has not been thoroughly investigated. Therefore, this research field holds significant promise and is worthy of further exploration.

Current evidence suggests that gut microbiota play a role in the occurrence and progression of AA. Therefore, maintaining the composition and stability of gut microbiota can be helpful in preventing and treating alopecia areata. However, the complex mechanisms through which these microbes influence AA require further clinical trials, molecular mechanism studies, and other research efforts to help us gain a more comprehensive understanding of the role of gut microbiota in the pathogenesis of alopecia areata. This will provide important scientific evidence for the development of new treatment strategies, which is also our next direction.

Due to the complex nature of factors influencing gut microbiota, traditional observational studies struggle to determine the causal relationship between gut microbiota and AA. Therefore, we employed Mendelian randomization, which effectively mitigates this limitation. Additionally, we utilized the latest gut microbiota data, enhancing the comprehensiveness and cutting-edge nature of our study. However, there are some limitations to note. Firstly, our sample size of AA patients is relatively small. Secondly, the GWAS data used in this study mainly come from European populations, which indeed limits the generalizability of our findings to other racial groups and requires further validation in subsequent research.

## Conclusion

We have identified causal relationships between 16 gut microbiota taxa and alopecia areata. However, further research is required to elucidate the specific mechanisms through which these microbiota influence alopecia areata.

## Data availability statement

The original contributions presented in this study are included in this article/Supplementary material, further inquiries can be directed to the corresponding authors.

## Ethics statement

All datasets utilized in this study are publicly available. Ethical approval and written informed consent were obtained as part of the original study.

## Author contributions

DB: Conceptualization, Writing – original draft. JT: Writing – original draft. DY: Data curation, Writing – review & editing. YC: Methodology, Writing – review & editing. MQ: Visualization, Writing – review & editing. JS: Funding acquisition, Resources, Writing – review & editing. SG: Funding acquisition, Supervision, Writing – review & editing.

## Footnotes

1
https://www.ebi.ac.uk/gwas/

2
https://r10.finngen.fi/
